# Bis(2,2-dimethyl-2,3-dihydro-1-benzofuran-7-yl) carbonate

**DOI:** 10.1107/S1600536811000341

**Published:** 2011-01-12

**Authors:** Lin-Tao Yang, Xian-Fu Luo, Ai-Xi Hu, Yu Wang

**Affiliations:** a College of Chemistry and Chemical Engineering, Hunan University, Changsha 410082, People’s Republic of China; b Hunan Research Institute of Chemical Industry, Changsha 410007, People’s Republic of China

## Abstract

The title compound, C_21_H_22_O_5_, crystallizes with three mol­ecules in the asymmetric unit. In one mol­ecule, two methyl groups are disordered over two positions with a site occupation factor of 0.72 (2) for the major occupancy site. The benzene rings make dihedral angles of 35.3 (6), 29.7 (6) and 40.6 (7)° in the three molecules.

## Related literature

The compound was obtained as a by-product during the derivatization of the commercially available carbofuran (systematic name 2,2-dimethyl-2,3-dihydro-1-benzofuran-7-yl methyl­carbamate), a popular carbamate insecticide. For background to insecticides, see: Tomlin (1994 ▶). For related structures, see: Xu *et al.* (2005 ▶); Li *et al.* (2009 ▶).
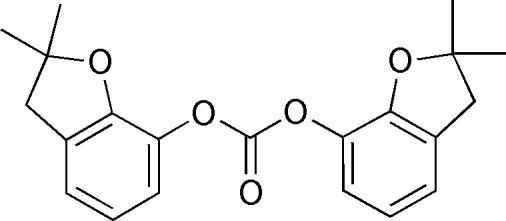


## Experimental

### 

#### Crystal data

C_21_H_22_O_5_
*M* *_r_* = 354.39
Orthorhombic, 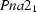

*a* = 16.6409 (6) Å
*b* = 23.2581 (9) Å
*c* = 14.5045 (6) Å
*V* = 5613.8 (4) Å^3^
*Z* = 12
Mo *K*α radiation
μ = 0.09 mm^−1^
*T* = 173 K
0.46 × 0.44 × 0.42 mm

#### Data collection

Bruker SMART 1000 CCD diffractometer
Absorption correction: multi-scan (*SADABS*; Sheldrick, 2004 ▶) *T*_min_ = 0.960, *T*_max_ = 0.964
27906 measured reflections
6368 independent reflections
4923 reflections with *I* > 2σ(*I*)
*R*_int_ = 0.030

#### Refinement

*R*[*F* ^2^ > 2σ(*F* ^2^)] = 0.042
*wR*(*F* ^2^) = 0.109
*S* = 1.06
6368 reflections
732 parameters
32 restraints
H-atom parameters constrained
Δρ_max_ = 0.23 e Å^−3^
Δρ_min_ = −0.21 e Å^−3^

### 

Data collection: *SMART* (Bruker, 2001 ▶); cell refinement: *SAINT-Plus* (Bruker, 2003 ▶); data reduction: *SAINT-Plus*; program(s) used to solve structure: *SHELXTL* (Sheldrick, 2008 ▶); program(s) used to refine structure: *SHELXTL*; molecular graphics: *SHELXTL*; software used to prepare material for publication: *SHELXTL*.

## Supplementary Material

Crystal structure: contains datablocks I, global. DOI: 10.1107/S1600536811000341/bt5448sup1.cif

Structure factors: contains datablocks I. DOI: 10.1107/S1600536811000341/bt5448Isup2.hkl

Additional supplementary materials:  crystallographic information; 3D view; checkCIF report

## Figures and Tables

**Fig. 1.**
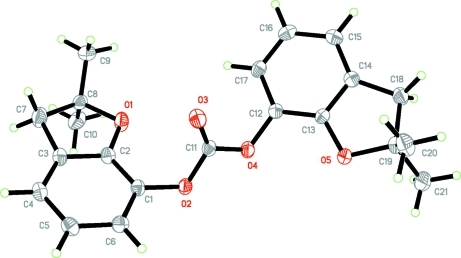
Perspective view of one the the three molecules of the title compound showing 30% probability displacement ellipsoids.

## supplementary crystallographic information

### Comment

Bis(2,2-dimethyldihydrobenzofuran-7-yl)carbonate(I), C_21_H_22_O_5_, was obtained as a by-product during the attempted preparation of the derivatives of the commercial Carbofuran (Xu  *et al.*, 2005; Li *et al.*, 2009), which is a popular carbamate insecticide (Tomlin, 1994). In the molecule of the title compound, all bond lengths and angles are within normal ranges. In the crystal, the dihedral angles between the phenyl rings of C1—C6 and C12—C17, C22—C27 and C33—C38, C43—C48 and C54—C59 are 35.3 (6)°, 29.7 (6)°, 40.6 (7)°. The crystal packing is stabilized by π···π interactions.

### Experimental

0.05 mol of bis(trichloromethyl)carbonate was dissolved in 200 mL toluene at room temperature, then the solution was cooled to 263.15 K, 1.0 mL triethylamine was added. After half an hour, the solution of 0.10 mol 2,2-dimethy-2,3-dihydrobenzofuran-7-ol in 100 mL toluene was added dropwise to give bis(2,2-dimethyldihydrobenzofuran-7-yl) carbonate as an amber solid of 14.3 g, yielded 80.5%. The solid was purified by recrystallization from saturated ethanol solution, giving the title compound as a colourless crystalline solid. Single crystals suitable for X-ray diffraction were obtained by slow evaporation of an ethyl acetate solution at room temperature over a period of five days. The identity of the title compound was confirmed by NMR and MS spectroscopy.

### Refinement

Methyl H atoms were placed in calculated positions, with C—H=0.96 Å, and torsion angles were refined, with Uiso(H)=1.5Ueq(C). Other H atoms were placed in geometrically idealized positions and refined as riding model, with N—H distance of 0.86 Å, C—H distances of 0.98Å (C3—H3), 0.93Å (aromatic H atoms) and 0.97Å (methylene H atoms). The constraint Uiso(H)=1.2Ueq(carrier) was applied. The   the components of the anisotropic displacement parameters in the direction of the bond are restrained to be equal within an effective standard deviation 0.005, and C28, C29 are restrained with effective standard deviation 0.005 to have the same Uij components. The Uij values of the C62, C62B, C63, C63B atoms are refined to behave approximately isotropic within the effective standard deviation 0.005.

### Crystal data

**Table:**

C_21_H_22_O_5_	*D*_x_ = 1.258 Mg m^−^^3^
*M**_r_* = 354.39	Melting point: 361.45 K
Orthorhombic, *P**n**a*2_1_	Mo *K*α radiation, λ = 0.71073 Å
Hall symbol: P 2c -2n	Cell parameters from 9606 reflections
*a* = 16.6409 (6) Å	θ = 2.1–26.0°
*b* = 23.2581 (9) Å	µ = 0.09 mm^−^^1^
*c* = 14.5045 (6) Å	*T* = 173 K
*V* = 5613.8 (4) Å^3^	Block, colourless
*Z* = 12	0.46 × 0.44 × 0.42 mm
*F*(000) = 2256	

### Data collection

**Table:**

Bruker SMART 1000 CCD diffractometer	6368 independent reflections
Radiation source: fine-focus sealed tube	4923 reflections with *I* > 2σ(*I*)
graphite	*R*_int_ = 0.030
ω scans	θ_max_ = 27.1°, θ_min_ = 1.5°
Absorption correction: multi-scan (*SADABS*; Sheldrick, 2004)	*h* = −16→21
*T*_min_ = 0.960, *T*_max_ = 0.964	*k* = −27→29
27906 measured reflections	*l* = −17→18

### Refinement

**Table:**

Refinement on *F*^2^	Primary atom site location: structure-invariant direct methods
Least-squares matrix: full	Secondary atom site location: difference Fourier map
*R*[*F*^2^ > 2σ(*F*^2^)] = 0.042	Hydrogen site location: inferred from neighbouring sites
*wR*(*F*^2^) = 0.109	H-atom parameters constrained
*S* = 1.06	*w* = 1/[σ^2^(*F*_o_^2^) + (0.0497*P*)^2^ + 1.3774*P*] where *P* = (*F*_o_^2^ + 2*F*_c_^2^)/3
6368 reflections	(Δ/σ)_max_ < 0.001
732 parameters	Δρ_max_ = 0.23 e Å^−^^3^
32 restraints	Δρ_min_ = −0.21 e Å^−^^3^

### Special details

**Table:**

Experimental. ^1^H NMR in CDCl_3_ (300 MHz), delta: 1.50(s,12H,4CH_3_), 3.05(s,4H,2CH_2_), 6.78–7.27(m,6H,2C_6_H_3_)LC-MS: 372.2(M+18)
Geometry. All esds (except the esd in the dihedral angle between two l.s. planes) are estimated using the full covariance matrix. The cell esds are taken into account individually in the estimation of esds in distances, angles and torsion angles; correlations between esds in cell parameters are only used when they are defined by crystal symmetry. An approximate (isotropic) treatment of cell esds is used for estimating esds involving l.s. planes.
Refinement. Refinement of *F*^2^ against ALL reflections. The weighted *R*-factor wR and goodness of fit *S* are based on *F*^2^, conventional *R*-factors *R* are based on *F*, with *F* set to zero for negative *F*^2^. The threshold expression of *F*^2^ > σ(*F*^2^) is used only for calculating *R*-factors(gt) etc. and is not relevant to the choice of reflections for refinement. *R*-factors based on *F*^2^ are statistically about twice as large as those based on *F*, and *R*- factors based on ALL data will be even larger.

### Fractional atomic coordinates and isotropic or equivalent isotropic displacement parameters (Å^2^)

**Table:**

	*x*	*y*	*z*	*U*_iso_*/*U*_eq_	Occ. (<1)
C1	0.72869 (16)	0.64804 (12)	0.2593 (2)	0.0317 (6)	
C2	0.77503 (17)	0.67717 (12)	0.1957 (2)	0.0339 (6)	
C3	0.81873 (17)	0.64739 (13)	0.1300 (2)	0.0367 (7)	
C4	0.81567 (17)	0.58778 (13)	0.1275 (2)	0.0383 (7)	
H4	0.8447	0.5670	0.0820	0.046*	
C5	0.76969 (18)	0.55890 (13)	0.1924 (2)	0.0403 (7)	
H5	0.7679	0.5181	0.1916	0.048*	
C6	0.72644 (17)	0.58870 (13)	0.2582 (2)	0.0375 (7)	
H6	0.6953	0.5684	0.3024	0.045*	
C7	0.8595 (2)	0.69120 (15)	0.0700 (3)	0.0518 (9)	
H7A	0.8345	0.6929	0.0082	0.062*	
H7B	0.9175	0.6826	0.0632	0.062*	
C8	0.84633 (17)	0.74798 (13)	0.1235 (2)	0.0363 (7)	
C9	0.8164 (2)	0.79648 (16)	0.0643 (2)	0.0551 (9)	
H9A	0.7684	0.7841	0.0307	0.083*	
H9B	0.8584	0.8075	0.0203	0.083*	
H9C	0.8031	0.8295	0.1034	0.083*	
C10	0.9177 (2)	0.76493 (18)	0.1789 (2)	0.0547 (9)	
H10A	0.9040	0.7979	0.2179	0.082*	
H10B	0.9618	0.7755	0.1374	0.082*	
H10C	0.9344	0.7326	0.2178	0.082*	
C11	0.63004 (16)	0.71444 (13)	0.2967 (2)	0.0320 (6)	
C12	0.55604 (16)	0.79348 (12)	0.34947 (19)	0.0310 (6)	
C13	0.48895 (16)	0.79984 (12)	0.40412 (19)	0.0303 (6)	
C14	0.43827 (17)	0.84686 (12)	0.3926 (2)	0.0349 (7)	
C15	0.45287 (19)	0.88649 (13)	0.3243 (2)	0.0405 (7)	
H15	0.4176	0.9181	0.3154	0.049*	
C16	0.52035 (19)	0.87953 (14)	0.2683 (2)	0.0424 (7)	
H16	0.5307	0.9064	0.2204	0.051*	
C17	0.57225 (18)	0.83401 (13)	0.2820 (2)	0.0370 (7)	
H17	0.6191	0.8305	0.2450	0.044*	
C18	0.37437 (19)	0.84320 (14)	0.4653 (2)	0.0440 (8)	
H18A	0.3203	0.8490	0.4385	0.053*	
H18B	0.3834	0.8720	0.5145	0.053*	
C19	0.38424 (18)	0.78166 (14)	0.5022 (2)	0.0379 (7)	
C20	0.3244 (2)	0.74160 (16)	0.4557 (3)	0.0544 (9)	
H20A	0.3299	0.7448	0.3886	0.082*	
H20B	0.2697	0.7523	0.4738	0.082*	
H20C	0.3351	0.7019	0.4747	0.082*	
C21	0.3823 (3)	0.77725 (19)	0.6050 (2)	0.0622 (10)	
H21A	0.3937	0.7376	0.6235	0.093*	
H21B	0.3290	0.7884	0.6275	0.093*	
H21C	0.4229	0.8029	0.6314	0.093*	
C22	0.50518 (17)	0.52554 (13)	0.3246 (2)	0.0355 (6)	
C23	0.45542 (19)	0.49810 (13)	0.3866 (2)	0.0397 (7)	
C24	0.4071 (2)	0.52908 (15)	0.4452 (2)	0.0495 (9)	
C25	0.4077 (2)	0.58836 (15)	0.4427 (3)	0.0513 (9)	
H25	0.3746	0.6099	0.4835	0.062*	
C26	0.4570 (2)	0.61611 (14)	0.3803 (3)	0.0476 (8)	
H26	0.4579	0.6569	0.3782	0.057*	
C27	0.50489 (19)	0.58482 (14)	0.3211 (2)	0.0424 (7)	
H27	0.5379	0.6042	0.2775	0.051*	
C28	0.3563 (3)	0.48691 (17)	0.4983 (3)	0.0785 (13)	
H28A	0.2992	0.4892	0.4792	0.094*	
H28B	0.3600	0.4939	0.5655	0.094*	
C29	0.3931 (2)	0.42830 (16)	0.4725 (3)	0.0560 (9)	
C30	0.4447 (3)	0.40512 (19)	0.5482 (3)	0.0719 (12)	
H30A	0.4758	0.3723	0.5252	0.108*	
H30B	0.4106	0.3926	0.5996	0.108*	
H30C	0.4815	0.4352	0.5696	0.108*	
C31	0.3326 (3)	0.3848 (2)	0.4381 (3)	0.0740 (12)	
H31A	0.3020	0.4014	0.3868	0.111*	
H31B	0.2958	0.3745	0.4882	0.111*	
H31C	0.3608	0.3503	0.4168	0.111*	
C32	0.60410 (16)	0.45748 (14)	0.2938 (2)	0.0380 (7)	
C33	0.67945 (18)	0.37786 (13)	0.2413 (2)	0.0378 (7)	
C34	0.74682 (17)	0.37333 (12)	0.1865 (2)	0.0343 (6)	
C35	0.79846 (19)	0.32660 (13)	0.1949 (2)	0.0399 (7)	
C36	0.7833 (2)	0.28498 (15)	0.2601 (2)	0.0494 (8)	
H36	0.8187	0.2533	0.2671	0.059*	
C37	0.7160 (2)	0.28993 (16)	0.3153 (3)	0.0552 (9)	
H37	0.7055	0.2615	0.3607	0.066*	
C38	0.6636 (2)	0.33572 (15)	0.3053 (2)	0.0486 (8)	
H38	0.6168	0.3381	0.3426	0.058*	
C39	0.8623 (2)	0.33340 (14)	0.1226 (3)	0.0484 (8)	
H39A	0.8531	0.3071	0.0701	0.058*	
H39B	0.9165	0.3262	0.1485	0.058*	
C40	0.85265 (18)	0.39687 (15)	0.0932 (2)	0.0421 (8)	
C41	0.90819 (19)	0.43531 (15)	0.1465 (3)	0.0496 (9)	
H41A	0.8983	0.4755	0.1294	0.074*	
H41B	0.9640	0.4252	0.1324	0.074*	
H41C	0.8985	0.4304	0.2127	0.074*	
C42	0.8569 (2)	0.40710 (19)	−0.0094 (2)	0.0607 (10)	
H42A	0.8183	0.3820	−0.0408	0.091*	
H42B	0.9113	0.3986	−0.0315	0.091*	
H42C	0.8438	0.4473	−0.0227	0.091*	
C43	0.0015 (2)	0.33792 (15)	0.3517 (3)	0.0486 (8)	
C44	−0.05123 (19)	0.36897 (14)	0.4055 (2)	0.0417 (7)	
C45	−0.0936 (2)	0.34370 (15)	0.4767 (3)	0.0500 (9)	
C46	−0.0841 (2)	0.28554 (18)	0.4923 (3)	0.0697 (14)	
H46	−0.1126	0.2673	0.5409	0.084*	
C47	−0.0327 (3)	0.25404 (18)	0.4367 (4)	0.0792 (15)	
H47	−0.0266	0.2139	0.4469	0.095*	
C48	0.0094 (2)	0.28026 (17)	0.3668 (3)	0.0665 (12)	
H48	0.0441	0.2581	0.3289	0.080*	
C49	−0.1459 (3)	0.38968 (18)	0.5191 (3)	0.0648 (11)	
H49A	−0.1391	0.3911	0.5868	0.078*	
H49B	−0.2033	0.3831	0.5046	0.078*	
C50	−0.11503 (19)	0.44496 (15)	0.4736 (2)	0.0431 (7)	
C51	−0.0575 (2)	0.47812 (19)	0.5336 (3)	0.0668 (11)	
H51A	−0.0155	0.4522	0.5563	0.100*	
H51B	−0.0867	0.4946	0.5860	0.100*	
H51C	−0.0330	0.5091	0.4975	0.100*	
C52	−0.1806 (2)	0.4841 (2)	0.4376 (4)	0.0790 (14)	
H52A	−0.1563	0.5171	0.4062	0.118*	
H52B	−0.2136	0.4976	0.4892	0.118*	
H52C	−0.2144	0.4628	0.3940	0.118*	
C53	0.10357 (19)	0.39918 (16)	0.3031 (3)	0.0469 (8)	
C54	0.19407 (19)	0.46089 (16)	0.2276 (2)	0.0481 (8)	
C55	0.2647 (2)	0.44617 (16)	0.1850 (2)	0.0471 (8)	
C56	0.3256 (2)	0.48610 (18)	0.1742 (3)	0.0543 (9)	
C57	0.3172 (3)	0.54066 (18)	0.2108 (3)	0.0647 (11)	
H57	0.3589	0.5683	0.2044	0.078*	
C58	0.2467 (3)	0.55435 (18)	0.2571 (3)	0.0679 (11)	
H58	0.2407	0.5913	0.2840	0.081*	
C59	0.1853 (2)	0.51498 (18)	0.2643 (3)	0.0616 (10)	
H59	0.1368	0.5252	0.2947	0.074*	
C60	0.3903 (2)	0.4579 (2)	0.1167 (4)	0.0740 (12)	
H60A	0.4438	0.4621	0.1458	0.089*	
H60B	0.3921	0.4745	0.0539	0.089*	
C61	0.3646 (2)	0.39495 (18)	0.1140 (3)	0.0551 (10)	
C62	0.3733 (10)	0.3503 (10)	0.0322 (12)	0.053 (5)	0.28 (2)
H62A	0.3470	0.3656	−0.0231	0.080*	0.28 (2)
H62B	0.3479	0.3139	0.0497	0.080*	0.28 (2)
H62C	0.4304	0.3438	0.0193	0.080*	0.28 (2)
C63	0.4118 (10)	0.3650 (7)	0.1904 (12)	0.035 (4)	0.28 (2)
H63A	0.4343	0.3939	0.2322	0.052*	0.28 (2)
H63B	0.4555	0.3425	0.1631	0.052*	0.28 (2)
H63C	0.3759	0.3394	0.2248	0.052*	0.28 (2)
C62B	0.3559 (5)	0.3804 (6)	0.0136 (5)	0.069 (2)	0.72 (2)
H62D	0.3247	0.4105	−0.0173	0.104*	0.72 (2)
H62E	0.3281	0.3435	0.0073	0.104*	0.72 (2)
H62F	0.4093	0.3778	−0.0147	0.104*	0.72 (2)
C63B	0.4127 (7)	0.3545 (6)	0.1693 (11)	0.109 (4)	0.72 (2)
H63D	0.4192	0.3697	0.2319	0.163*	0.72 (2)
H63E	0.4657	0.3496	0.1408	0.163*	0.72 (2)
H63F	0.3853	0.3172	0.1720	0.163*	0.72 (2)
O1	0.78087 (13)	0.73538 (9)	0.19029 (16)	0.0444 (5)	
O2	0.68790 (11)	0.67934 (8)	0.32817 (13)	0.0346 (4)	
O3	0.60238 (12)	0.71474 (10)	0.22166 (15)	0.0428 (5)	
O4	0.60936 (11)	0.74815 (9)	0.36804 (14)	0.0357 (5)	
O5	0.46542 (12)	0.76326 (9)	0.47200 (14)	0.0372 (5)	
O6	0.44698 (14)	0.44023 (9)	0.39277 (18)	0.0533 (6)	
O7	0.54932 (12)	0.49397 (9)	0.25984 (15)	0.0398 (5)	
O8	0.62912 (14)	0.45562 (11)	0.37023 (18)	0.0556 (7)	
O9	0.62560 (12)	0.42365 (10)	0.22312 (15)	0.0411 (5)	
O10	0.76908 (12)	0.41200 (9)	0.12079 (15)	0.0395 (5)	
O11	−0.06665 (14)	0.42617 (9)	0.39410 (15)	0.0472 (6)	
O12	0.04291 (14)	0.36401 (11)	0.27900 (16)	0.0506 (6)	
O13	0.12750 (16)	0.40829 (13)	0.37863 (18)	0.0665 (8)	
O14	0.12937 (14)	0.42191 (12)	0.22380 (17)	0.0538 (6)	
O15	0.28074 (13)	0.39351 (12)	0.1484 (2)	0.0617 (7)	

### Atomic displacement parameters (Å^2^)

**Table:**

	*U*^11^	*U*^22^	*U*^33^	*U*^12^	*U*^13^	*U*^23^
C1	0.0255 (13)	0.0375 (16)	0.0320 (14)	0.0011 (12)	−0.0016 (12)	−0.0040 (13)
C2	0.0291 (14)	0.0344 (15)	0.0382 (16)	−0.0030 (12)	0.0014 (12)	−0.0035 (13)
C3	0.0312 (15)	0.0416 (17)	0.0375 (16)	0.0018 (12)	0.0024 (13)	−0.0056 (14)
C4	0.0339 (15)	0.0412 (17)	0.0398 (17)	0.0045 (13)	−0.0004 (13)	−0.0089 (14)
C5	0.0371 (16)	0.0335 (16)	0.0502 (19)	−0.0011 (13)	−0.0031 (15)	−0.0057 (14)
C6	0.0284 (14)	0.0409 (17)	0.0433 (16)	−0.0037 (13)	0.0004 (13)	0.0045 (14)
C7	0.052 (2)	0.050 (2)	0.054 (2)	−0.0026 (16)	0.0206 (17)	−0.0017 (17)
C8	0.0320 (15)	0.0419 (17)	0.0351 (16)	−0.0023 (13)	0.0054 (13)	0.0048 (13)
C9	0.057 (2)	0.066 (2)	0.042 (2)	0.0163 (18)	−0.0035 (17)	0.0103 (18)
C10	0.0382 (18)	0.086 (3)	0.0402 (19)	−0.0060 (17)	−0.0047 (15)	0.0029 (18)
C11	0.0245 (13)	0.0387 (16)	0.0328 (15)	−0.0030 (12)	0.0024 (12)	0.0002 (12)
C12	0.0285 (14)	0.0366 (15)	0.0279 (14)	−0.0007 (12)	−0.0036 (11)	−0.0036 (12)
C13	0.0326 (15)	0.0322 (15)	0.0263 (13)	−0.0043 (11)	−0.0014 (12)	−0.0028 (12)
C14	0.0349 (15)	0.0372 (16)	0.0327 (15)	0.0006 (12)	−0.0038 (12)	−0.0053 (13)
C15	0.0441 (17)	0.0346 (16)	0.0427 (18)	0.0029 (13)	−0.0062 (14)	0.0003 (14)
C16	0.0485 (18)	0.0437 (18)	0.0349 (16)	−0.0089 (14)	−0.0015 (15)	0.0072 (14)
C17	0.0347 (15)	0.0453 (18)	0.0308 (16)	−0.0064 (13)	0.0006 (13)	−0.0027 (13)
C18	0.0412 (17)	0.0404 (17)	0.050 (2)	0.0054 (14)	0.0057 (15)	−0.0005 (15)
C19	0.0356 (16)	0.0409 (17)	0.0373 (17)	0.0045 (13)	0.0071 (13)	−0.0019 (13)
C20	0.0396 (18)	0.047 (2)	0.076 (3)	0.0026 (15)	0.0025 (18)	−0.0062 (18)
C21	0.070 (2)	0.077 (3)	0.040 (2)	0.021 (2)	0.0151 (18)	0.0024 (18)
C22	0.0280 (14)	0.0436 (17)	0.0350 (16)	0.0030 (13)	0.0001 (12)	−0.0049 (13)
C23	0.0397 (17)	0.0391 (17)	0.0402 (17)	0.0094 (13)	0.0037 (14)	−0.0011 (14)
C24	0.056 (2)	0.045 (2)	0.048 (2)	0.0147 (16)	0.0200 (17)	0.0056 (15)
C25	0.060 (2)	0.0437 (19)	0.050 (2)	0.0167 (16)	0.0109 (17)	−0.0023 (15)
C26	0.0496 (19)	0.0375 (17)	0.056 (2)	0.0040 (15)	−0.0028 (17)	−0.0037 (16)
C27	0.0334 (15)	0.0461 (18)	0.0478 (19)	−0.0041 (14)	−0.0007 (14)	0.0004 (15)
C28	0.095 (3)	0.055 (2)	0.085 (3)	0.025 (2)	0.054 (2)	0.016 (2)
C29	0.064 (2)	0.0441 (18)	0.060 (2)	0.0113 (16)	0.0285 (19)	0.0100 (17)
C30	0.104 (3)	0.064 (3)	0.047 (2)	0.013 (2)	−0.004 (2)	−0.0098 (19)
C31	0.054 (2)	0.098 (3)	0.070 (3)	−0.004 (2)	0.016 (2)	0.010 (2)
C32	0.0223 (13)	0.0500 (19)	0.0416 (18)	−0.0038 (13)	0.0041 (13)	−0.0125 (14)
C33	0.0365 (16)	0.0415 (17)	0.0356 (17)	−0.0006 (13)	0.0046 (13)	−0.0066 (13)
C34	0.0359 (15)	0.0378 (16)	0.0293 (15)	−0.0016 (12)	0.0004 (12)	−0.0029 (13)
C35	0.0413 (17)	0.0375 (16)	0.0409 (17)	0.0008 (13)	0.0014 (14)	−0.0037 (14)
C36	0.060 (2)	0.0440 (18)	0.0442 (18)	0.0050 (16)	−0.0014 (17)	0.0016 (16)
C37	0.074 (3)	0.050 (2)	0.0415 (19)	−0.0058 (18)	0.0062 (18)	0.0059 (16)
C38	0.054 (2)	0.048 (2)	0.0435 (19)	−0.0087 (16)	0.0151 (16)	−0.0030 (15)
C39	0.0439 (18)	0.0465 (19)	0.055 (2)	0.0133 (15)	0.0085 (16)	−0.0051 (17)
C40	0.0341 (16)	0.053 (2)	0.0394 (17)	0.0124 (14)	0.0111 (14)	0.0040 (14)
C41	0.0372 (17)	0.052 (2)	0.060 (2)	0.0065 (15)	0.0063 (16)	0.0094 (17)
C42	0.059 (2)	0.080 (3)	0.042 (2)	0.025 (2)	0.0153 (18)	0.0029 (18)
C43	0.0464 (18)	0.0462 (19)	0.053 (2)	0.0001 (16)	−0.0135 (17)	−0.0018 (16)
C44	0.0447 (18)	0.0428 (18)	0.0376 (17)	−0.0077 (14)	−0.0052 (14)	0.0041 (14)
C45	0.0479 (19)	0.055 (2)	0.0468 (19)	−0.0177 (16)	−0.0089 (16)	0.0155 (17)
C46	0.053 (2)	0.068 (3)	0.088 (3)	−0.026 (2)	−0.027 (2)	0.043 (2)
C47	0.058 (2)	0.048 (2)	0.132 (5)	−0.001 (2)	−0.034 (3)	0.021 (3)
C48	0.050 (2)	0.052 (2)	0.097 (3)	0.0035 (18)	−0.020 (2)	−0.001 (2)
C49	0.069 (2)	0.076 (3)	0.050 (2)	−0.027 (2)	0.015 (2)	0.0076 (19)
C50	0.0385 (17)	0.057 (2)	0.0342 (16)	−0.0065 (15)	0.0076 (14)	0.0002 (15)
C51	0.073 (3)	0.083 (3)	0.044 (2)	−0.028 (2)	0.011 (2)	−0.017 (2)
C52	0.047 (2)	0.107 (4)	0.084 (3)	0.010 (2)	0.011 (2)	0.025 (3)
C53	0.0340 (17)	0.061 (2)	0.046 (2)	0.0074 (15)	−0.0052 (16)	−0.0036 (17)
C54	0.0423 (18)	0.070 (2)	0.0320 (16)	−0.0017 (17)	−0.0059 (14)	−0.0007 (16)
C55	0.0445 (18)	0.064 (2)	0.0333 (16)	−0.0038 (16)	−0.0061 (15)	−0.0047 (16)
C56	0.0462 (19)	0.070 (2)	0.046 (2)	−0.0087 (18)	−0.0104 (17)	−0.0001 (18)
C57	0.068 (3)	0.060 (2)	0.067 (3)	−0.005 (2)	−0.024 (2)	0.002 (2)
C58	0.076 (3)	0.058 (2)	0.070 (3)	0.014 (2)	−0.020 (2)	−0.003 (2)
C59	0.061 (2)	0.075 (3)	0.049 (2)	0.022 (2)	−0.0134 (19)	−0.004 (2)
C60	0.052 (2)	0.090 (3)	0.079 (3)	−0.023 (2)	0.008 (2)	−0.010 (3)
C61	0.0402 (18)	0.083 (3)	0.042 (2)	−0.0155 (17)	0.0100 (16)	−0.0219 (19)
C62	0.053 (6)	0.061 (7)	0.046 (6)	−0.004 (4)	0.006 (4)	−0.016 (4)
C63	0.028 (5)	0.033 (5)	0.043 (5)	−0.010 (4)	0.007 (4)	−0.016 (4)
C62B	0.065 (3)	0.090 (5)	0.053 (3)	−0.017 (3)	0.007 (3)	−0.014 (3)
C63B	0.092 (5)	0.120 (6)	0.114 (6)	0.002 (4)	0.007 (4)	0.008 (4)
O1	0.0434 (12)	0.0377 (12)	0.0521 (14)	−0.0032 (9)	0.0185 (11)	−0.0066 (10)
O2	0.0298 (10)	0.0430 (12)	0.0310 (10)	0.0040 (9)	0.0036 (9)	−0.0001 (9)
O3	0.0373 (11)	0.0540 (13)	0.0371 (12)	0.0072 (10)	−0.0085 (10)	−0.0111 (10)
O4	0.0310 (10)	0.0451 (12)	0.0311 (11)	0.0045 (9)	0.0020 (9)	−0.0036 (9)
O5	0.0360 (11)	0.0422 (12)	0.0335 (11)	0.0050 (9)	0.0058 (9)	0.0032 (9)
O6	0.0556 (14)	0.0377 (12)	0.0667 (17)	0.0113 (10)	0.0289 (13)	0.0022 (12)
O7	0.0325 (11)	0.0500 (13)	0.0368 (11)	0.0023 (9)	0.0072 (9)	−0.0049 (10)
O8	0.0452 (13)	0.0713 (17)	0.0502 (15)	0.0167 (12)	−0.0141 (11)	−0.0236 (13)
O9	0.0382 (11)	0.0488 (13)	0.0364 (11)	0.0039 (10)	0.0084 (9)	−0.0042 (10)
O10	0.0349 (11)	0.0450 (12)	0.0387 (11)	0.0100 (9)	0.0098 (9)	0.0033 (10)
O11	0.0611 (14)	0.0405 (12)	0.0399 (13)	−0.0074 (11)	0.0163 (11)	0.0020 (10)
O12	0.0430 (13)	0.0650 (15)	0.0439 (14)	0.0032 (11)	−0.0005 (11)	−0.0105 (12)
O13	0.0603 (16)	0.099 (2)	0.0406 (14)	−0.0197 (15)	−0.0164 (13)	0.0104 (14)
O14	0.0416 (13)	0.0870 (19)	0.0329 (12)	−0.0041 (12)	0.0019 (10)	−0.0061 (12)
O15	0.0396 (13)	0.0787 (18)	0.0669 (17)	−0.0182 (12)	0.0151 (12)	−0.0296 (14)

### Geometric parameters (Å, °)

**Table:**

C1—C2	1.380 (4)	C34—O10	1.362 (4)
C1—C6	1.381 (4)	C34—C35	1.391 (4)
C1—O2	1.410 (3)	C35—C36	1.377 (5)
C2—O1	1.360 (4)	C35—C39	1.501 (5)
C2—C3	1.385 (4)	C36—C37	1.381 (5)
C3—C4	1.388 (4)	C36—H36	0.9500
C3—C7	1.502 (5)	C37—C38	1.384 (5)
C4—C5	1.387 (4)	C37—H37	0.9500
C4—H4	0.9500	C38—H38	0.9500
C5—C6	1.381 (4)	C39—C40	1.545 (5)
C5—H5	0.9500	C39—H39A	0.9900
C6—H6	0.9500	C39—H39B	0.9900
C7—C8	1.547 (5)	C40—O10	1.489 (3)
C7—H7A	0.9900	C40—C41	1.501 (5)
C7—H7B	0.9900	C40—C42	1.509 (5)
C8—O1	1.487 (4)	C41—H41A	0.9800
C8—C10	1.487 (4)	C41—H41B	0.9800
C8—C9	1.502 (4)	C41—H41C	0.9800
C9—H9A	0.9800	C42—H42A	0.9800
C9—H9B	0.9800	C42—H42B	0.9800
C9—H9C	0.9800	C42—H42C	0.9800
C10—H10A	0.9800	C43—C48	1.365 (5)
C10—H10B	0.9800	C43—C44	1.378 (5)
C10—H10C	0.9800	C43—O12	1.398 (4)
C11—O3	1.182 (4)	C44—O11	1.365 (4)
C11—O2	1.342 (3)	C44—C45	1.382 (5)
C11—O4	1.343 (4)	C45—C46	1.381 (5)
C12—C13	1.377 (4)	C45—C49	1.510 (6)
C12—C17	1.386 (4)	C46—C47	1.385 (7)
C12—O4	1.404 (3)	C46—H46	0.9500
C13—O5	1.359 (3)	C47—C48	1.375 (7)
C13—C14	1.391 (4)	C47—H47	0.9500
C14—C15	1.375 (4)	C48—H48	0.9500
C14—C18	1.500 (4)	C49—C50	1.534 (5)
C15—C16	1.395 (5)	C49—H49A	0.9900
C15—H15	0.9500	C49—H49B	0.9900
C16—C17	1.380 (4)	C50—O11	1.473 (4)
C16—H16	0.9500	C50—C51	1.506 (5)
C17—H17	0.9500	C50—C52	1.514 (5)
C18—C19	1.537 (4)	C51—H51A	0.9800
C18—H18A	0.9900	C51—H51B	0.9800
C18—H18B	0.9900	C51—H51C	0.9800
C19—O5	1.483 (4)	C52—H52A	0.9800
C19—C21	1.495 (5)	C52—H52B	0.9800
C19—C20	1.521 (5)	C52—H52C	0.9800
C20—H20A	0.9800	C53—O13	1.185 (4)
C20—H20B	0.9800	C53—O14	1.336 (4)
C20—H20C	0.9800	C53—O12	1.346 (4)
C21—H21A	0.9800	C54—C55	1.371 (5)
C21—H21B	0.9800	C54—C59	1.374 (5)
C21—H21C	0.9800	C54—O14	1.409 (4)
C22—C23	1.379 (4)	C55—O15	1.361 (4)
C22—C27	1.380 (4)	C55—C56	1.384 (5)
C22—O7	1.401 (3)	C56—C57	1.382 (6)
C23—O6	1.356 (4)	C56—C60	1.512 (6)
C23—C24	1.374 (4)	C57—C58	1.389 (6)
C24—C25	1.379 (5)	C57—H57	0.9500
C24—C28	1.507 (5)	C58—C59	1.375 (6)
C25—C26	1.381 (5)	C58—H58	0.9500
C25—H25	0.9500	C59—H59	0.9500
C26—C27	1.380 (5)	C60—C61	1.525 (6)
C26—H26	0.9500	C60—H60A	0.9900
C27—H27	0.9500	C60—H60B	0.9900
C28—C29	1.540 (5)	C61—C63B	1.474 (13)
C28—H28A	0.9900	C61—O15	1.482 (4)
C28—H28B	0.9900	C61—C62B	1.501 (7)
C29—O6	1.490 (4)	C61—C63	1.527 (19)
C29—C30	1.495 (6)	C61—C62	1.584 (15)
C29—C31	1.512 (6)	C62—H62A	0.9800
C30—H30A	0.9800	C62—H62B	0.9800
C30—H30B	0.9800	C62—H62C	0.9800
C30—H30C	0.9800	C63—H63A	0.9800
C31—H31A	0.9800	C63—H63B	0.9800
C31—H31B	0.9800	C63—H63C	0.9800
C31—H31C	0.9800	C62B—H62D	0.9800
C32—O8	1.184 (4)	C62B—H62E	0.9800
C32—O7	1.340 (4)	C62B—H62F	0.9800
C32—O9	1.341 (4)	C63B—H63D	0.9800
C33—C38	1.375 (4)	C63B—H63E	0.9800
C33—C34	1.378 (4)	C63B—H63F	0.9800
C33—O9	1.417 (4)		
			
C2—C1—C6	119.8 (3)	C38—C37—H37	119.5
C2—C1—O2	119.3 (2)	C33—C38—C37	119.9 (3)
C6—C1—O2	120.8 (3)	C33—C38—H38	120.1
O1—C2—C1	124.6 (3)	C37—C38—H38	120.1
O1—C2—C3	114.9 (3)	C35—C39—C40	102.7 (2)
C1—C2—C3	120.6 (3)	C35—C39—H39A	111.2
C2—C3—C4	119.9 (3)	C40—C39—H39A	111.2
C2—C3—C7	107.3 (3)	C35—C39—H39B	111.2
C4—C3—C7	132.8 (3)	C40—C39—H39B	111.2
C5—C4—C3	119.1 (3)	H39A—C39—H39B	109.1
C5—C4—H4	120.4	O10—C40—C41	107.2 (3)
C3—C4—H4	120.4	O10—C40—C42	105.8 (3)
C6—C5—C4	120.9 (3)	C41—C40—C42	112.7 (3)
C6—C5—H5	119.6	O10—C40—C39	104.4 (2)
C4—C5—H5	119.6	C41—C40—C39	111.2 (3)
C1—C6—C5	119.7 (3)	C42—C40—C39	114.7 (3)
C1—C6—H6	120.1	C40—C41—H41A	109.5
C5—C6—H6	120.1	C40—C41—H41B	109.5
C3—C7—C8	103.0 (3)	H41A—C41—H41B	109.5
C3—C7—H7A	111.2	C40—C41—H41C	109.5
C8—C7—H7A	111.2	H41A—C41—H41C	109.5
C3—C7—H7B	111.2	H41B—C41—H41C	109.5
C8—C7—H7B	111.2	C40—C42—H42A	109.5
H7A—C7—H7B	109.1	C40—C42—H42B	109.5
O1—C8—C10	106.6 (3)	H42A—C42—H42B	109.5
O1—C8—C9	106.1 (2)	C40—C42—H42C	109.5
C10—C8—C9	112.0 (3)	H42A—C42—H42C	109.5
O1—C8—C7	105.2 (2)	H42B—C42—H42C	109.5
C10—C8—C7	112.6 (3)	C48—C43—C44	119.0 (4)
C9—C8—C7	113.7 (3)	C48—C43—O12	120.0 (4)
C8—C9—H9A	109.5	C44—C43—O12	120.9 (3)
C8—C9—H9B	109.5	O11—C44—C43	124.2 (3)
H9A—C9—H9B	109.5	O11—C44—C45	114.1 (3)
C8—C9—H9C	109.5	C43—C44—C45	121.7 (3)
H9A—C9—H9C	109.5	C46—C45—C44	118.8 (4)
H9B—C9—H9C	109.5	C46—C45—C49	133.9 (4)
C8—C10—H10A	109.5	C44—C45—C49	107.3 (3)
C8—C10—H10B	109.5	C45—C46—C47	119.6 (4)
H10A—C10—H10B	109.5	C45—C46—H46	120.2
C8—C10—H10C	109.5	C47—C46—H46	120.2
H10A—C10—H10C	109.5	C48—C47—C46	120.6 (4)
H10B—C10—H10C	109.5	C48—C47—H47	119.7
O3—C11—O2	126.6 (3)	C46—C47—H47	119.7
O3—C11—O4	127.3 (3)	C43—C48—C47	120.3 (4)
O2—C11—O4	106.1 (2)	C43—C48—H48	119.8
C13—C12—C17	119.4 (3)	C47—C48—H48	119.8
C13—C12—O4	118.8 (3)	C45—C49—C50	103.0 (3)
C17—C12—O4	121.6 (2)	C45—C49—H49A	111.2
O5—C13—C12	125.7 (3)	C50—C49—H49A	111.2
O5—C13—C14	113.8 (2)	C45—C49—H49B	111.2
C12—C13—C14	120.5 (3)	C50—C49—H49B	111.2
C15—C14—C13	120.4 (3)	H49A—C49—H49B	109.1
C15—C14—C18	132.1 (3)	O11—C50—C51	104.9 (3)
C13—C14—C18	107.5 (3)	O11—C50—C52	107.6 (3)
C14—C15—C16	118.9 (3)	C51—C50—C52	110.5 (4)
C14—C15—H15	120.5	O11—C50—C49	105.7 (3)
C16—C15—H15	120.5	C51—C50—C49	113.2 (3)
C17—C16—C15	120.6 (3)	C52—C50—C49	114.2 (3)
C17—C16—H16	119.7	C50—C51—H51A	109.5
C15—C16—H16	119.7	C50—C51—H51B	109.5
C16—C17—C12	120.1 (3)	H51A—C51—H51B	109.5
C16—C17—H17	120.0	C50—C51—H51C	109.5
C12—C17—H17	120.0	H51A—C51—H51C	109.5
C14—C18—C19	102.8 (2)	H51B—C51—H51C	109.5
C14—C18—H18A	111.2	C50—C52—H52A	109.5
C19—C18—H18A	111.2	C50—C52—H52B	109.5
C14—C18—H18B	111.2	H52A—C52—H52B	109.5
C19—C18—H18B	111.2	C50—C52—H52C	109.5
H18A—C18—H18B	109.1	H52A—C52—H52C	109.5
O5—C19—C21	107.2 (3)	H52B—C52—H52C	109.5
O5—C19—C20	106.7 (2)	O13—C53—O14	128.1 (3)
C21—C19—C20	112.7 (3)	O13—C53—O12	126.9 (3)
O5—C19—C18	105.2 (2)	O14—C53—O12	104.9 (3)
C21—C19—C18	114.2 (3)	C55—C54—C59	119.6 (3)
C20—C19—C18	110.2 (3)	C55—C54—O14	118.5 (3)
C19—C20—H20A	109.5	C59—C54—O14	121.6 (3)
C19—C20—H20B	109.5	O15—C55—C54	124.6 (3)
H20A—C20—H20B	109.5	O15—C55—C56	114.6 (3)
C19—C20—H20C	109.5	C54—C55—C56	120.8 (3)
H20A—C20—H20C	109.5	C57—C56—C55	119.9 (4)
H20B—C20—H20C	109.5	C57—C56—C60	133.1 (4)
C19—C21—H21A	109.5	C55—C56—C60	107.0 (3)
C19—C21—H21B	109.5	C56—C57—C58	118.8 (4)
H21A—C21—H21B	109.5	C56—C57—H57	120.6
C19—C21—H21C	109.5	C58—C57—H57	120.6
H21A—C21—H21C	109.5	C59—C58—C57	120.7 (4)
H21B—C21—H21C	109.5	C59—C58—H58	119.6
C23—C22—C27	119.0 (3)	C57—C58—H58	119.6
C23—C22—O7	120.6 (3)	C54—C59—C58	120.1 (4)
C27—C22—O7	120.0 (3)	C54—C59—H59	119.9
O6—C23—C24	114.8 (3)	C58—C59—H59	119.9
O6—C23—C22	124.4 (3)	C56—C60—C61	103.4 (3)
C24—C23—C22	120.8 (3)	C56—C60—H60A	111.1
C23—C24—C25	120.2 (3)	C61—C60—H60A	111.1
C23—C24—C28	107.7 (3)	C56—C60—H60B	111.1
C25—C24—C28	131.9 (3)	C61—C60—H60B	111.1
C24—C25—C26	119.2 (3)	H60A—C60—H60B	109.1
C24—C25—H25	120.4	C63B—C61—O15	108.3 (6)
C26—C25—H25	120.4	C63B—C61—C62B	115.9 (6)
C27—C26—C25	120.3 (3)	O15—C61—C62B	103.4 (4)
C27—C26—H26	119.8	C63B—C61—C60	116.5 (6)
C25—C26—H26	119.8	O15—C61—C60	106.1 (3)
C22—C27—C26	120.4 (3)	C62B—C61—C60	105.5 (6)
C22—C27—H27	119.8	O15—C61—C63	103.3 (7)
C26—C27—H27	119.8	C62B—C61—C63	130.6 (8)
C24—C28—C29	103.2 (3)	C60—C61—C63	105.9 (6)
C24—C28—H28A	111.1	C63B—C61—C62	86.5 (8)
C29—C28—H28A	111.1	O15—C61—C62	108.9 (6)
C24—C28—H28B	111.1	C60—C61—C62	128.5 (9)
C29—C28—H28B	111.1	C63—C61—C62	101.4 (11)
H28A—C28—H28B	109.1	C61—C62—H62A	109.5
O6—C29—C30	107.0 (3)	C61—C62—H62B	109.5
O6—C29—C31	105.6 (3)	H62A—C62—H62B	109.5
C30—C29—C31	112.6 (3)	C61—C62—H62C	109.5
O6—C29—C28	105.3 (3)	H62A—C62—H62C	109.5
C30—C29—C28	111.7 (4)	H62B—C62—H62C	109.5
C31—C29—C28	114.1 (4)	C61—C63—H63A	109.5
C29—C30—H30A	109.5	C61—C63—H63B	109.5
C29—C30—H30B	109.5	H63A—C63—H63B	109.5
H30A—C30—H30B	109.5	C61—C63—H63C	109.5
C29—C30—H30C	109.5	H63A—C63—H63C	109.5
H30A—C30—H30C	109.5	H63B—C63—H63C	109.5
H30B—C30—H30C	109.5	C61—C62B—H62D	109.5
C29—C31—H31A	109.5	C61—C62B—H62E	109.5
C29—C31—H31B	109.5	H62D—C62B—H62E	109.5
H31A—C31—H31B	109.5	C61—C62B—H62F	109.5
C29—C31—H31C	109.5	H62D—C62B—H62F	109.5
H31A—C31—H31C	109.5	H62E—C62B—H62F	109.5
H31B—C31—H31C	109.5	C61—C63B—H63D	109.5
O8—C32—O7	127.3 (3)	C61—C63B—H63E	109.5
O8—C32—O9	126.9 (3)	H63D—C63B—H63E	109.5
O7—C32—O9	105.8 (3)	C61—C63B—H63F	109.5
C38—C33—C34	119.4 (3)	H63D—C63B—H63F	109.5
C38—C33—O9	122.7 (3)	H63E—C63B—H63F	109.5
C34—C33—O9	117.7 (3)	C2—O1—C8	106.6 (2)
O10—C34—C33	125.1 (3)	C11—O2—C1	114.8 (2)
O10—C34—C35	114.2 (3)	C11—O4—C12	116.9 (2)
C33—C34—C35	120.8 (3)	C13—O5—C19	107.2 (2)
C36—C35—C34	119.8 (3)	C23—O6—C29	107.4 (2)
C36—C35—C39	133.1 (3)	C32—O7—C22	116.2 (2)
C34—C35—C39	107.1 (3)	C32—O9—C33	117.8 (2)
C35—C36—C37	119.2 (3)	C34—O10—C40	106.6 (2)
C35—C36—H36	120.4	C44—O11—C50	107.3 (2)
C37—C36—H36	120.4	C53—O12—C43	116.0 (3)
C36—C37—C38	121.0 (3)	C53—O14—C54	117.8 (3)
C36—C37—H37	119.5	C55—O15—C61	107.2 (3)
			
C6—C1—C2—O1	179.3 (3)	C45—C46—C47—C48	0.7 (6)
O2—C1—C2—O1	−4.3 (4)	C44—C43—C48—C47	−2.1 (6)
C6—C1—C2—C3	0.7 (4)	O12—C43—C48—C47	−178.4 (3)
O2—C1—C2—C3	177.0 (2)	C46—C47—C48—C43	0.4 (6)
O1—C2—C3—C4	−178.4 (3)	C46—C45—C49—C50	173.2 (4)
C1—C2—C3—C4	0.4 (4)	C44—C45—C49—C50	−10.0 (4)
O1—C2—C3—C7	−0.9 (4)	C45—C49—C50—O11	15.1 (4)
C1—C2—C3—C7	177.9 (3)	C45—C49—C50—C51	−99.2 (4)
C2—C3—C4—C5	−1.1 (4)	C45—C49—C50—C52	133.2 (4)
C7—C3—C4—C5	−177.9 (3)	C59—C54—C55—O15	178.3 (3)
C3—C4—C5—C6	0.8 (5)	O14—C54—C55—O15	−8.3 (5)
C2—C1—C6—C5	−0.9 (4)	C59—C54—C55—C56	−3.5 (5)
O2—C1—C6—C5	−177.2 (3)	O14—C54—C55—C56	169.9 (3)
C4—C5—C6—C1	0.2 (5)	O15—C55—C56—C57	−178.2 (3)
C2—C3—C7—C8	11.0 (3)	C54—C55—C56—C57	3.3 (5)
C4—C3—C7—C8	−171.8 (3)	O15—C55—C56—C60	4.2 (4)
C3—C7—C8—O1	−16.6 (3)	C54—C55—C56—C60	−174.3 (3)
C3—C7—C8—C10	99.1 (3)	C55—C56—C57—C58	−0.7 (6)
C3—C7—C8—C9	−132.3 (3)	C60—C56—C57—C58	176.2 (4)
C17—C12—C13—O5	179.3 (3)	C56—C57—C58—C59	−1.8 (6)
O4—C12—C13—O5	−5.1 (4)	C55—C54—C59—C58	1.0 (6)
C17—C12—C13—C14	−0.8 (4)	O14—C54—C59—C58	−172.2 (3)
O4—C12—C13—C14	174.8 (2)	C57—C58—C59—C54	1.7 (6)
O5—C13—C14—C15	−177.7 (3)	C57—C56—C60—C61	172.2 (4)
C12—C13—C14—C15	2.4 (4)	C55—C56—C60—C61	−10.7 (4)
O5—C13—C14—C18	3.2 (3)	C56—C60—C61—C63B	−107.4 (7)
C12—C13—C14—C18	−176.7 (3)	C56—C60—C61—O15	13.2 (4)
C13—C14—C15—C16	−1.6 (4)	C56—C60—C61—C62B	122.5 (5)
C18—C14—C15—C16	177.3 (3)	C56—C60—C61—C63	−96.2 (7)
C14—C15—C16—C17	−0.8 (5)	C56—C60—C61—C62	144.6 (10)
C15—C16—C17—C12	2.4 (5)	C1—C2—O1—C8	170.9 (3)
C13—C12—C17—C16	−1.6 (4)	C3—C2—O1—C8	−10.3 (3)
O4—C12—C17—C16	−177.0 (3)	C10—C8—O1—C2	−103.1 (3)
C15—C14—C18—C19	167.9 (3)	C9—C8—O1—C2	137.4 (3)
C13—C14—C18—C19	−13.1 (3)	C7—C8—O1—C2	16.6 (3)
C14—C18—C19—O5	17.8 (3)	O3—C11—O2—C1	12.7 (4)
C14—C18—C19—C21	135.0 (3)	O4—C11—O2—C1	−168.3 (2)
C14—C18—C19—C20	−96.9 (3)	C2—C1—O2—C11	65.4 (3)
C27—C22—C23—O6	176.0 (3)	C6—C1—O2—C11	−118.3 (3)
O7—C22—C23—O6	2.4 (5)	O3—C11—O4—C12	−8.4 (4)
C27—C22—C23—C24	−1.1 (5)	O2—C11—O4—C12	172.7 (2)
O7—C22—C23—C24	−174.7 (3)	C13—C12—O4—C11	129.4 (3)
O6—C23—C24—C25	−177.3 (3)	C17—C12—O4—C11	−55.2 (4)
C22—C23—C24—C25	0.1 (5)	C12—C13—O5—C19	−171.2 (3)
O6—C23—C24—C28	−1.6 (5)	C14—C13—O5—C19	8.8 (3)
C22—C23—C24—C28	175.8 (3)	C21—C19—O5—C13	−138.6 (3)
C23—C24—C25—C26	0.4 (6)	C20—C19—O5—C13	100.4 (3)
C28—C24—C25—C26	−174.1 (4)	C18—C19—O5—C13	−16.7 (3)
C24—C25—C26—C27	0.1 (5)	C24—C23—O6—C29	−7.1 (4)
C23—C22—C27—C26	1.7 (5)	C22—C23—O6—C29	175.6 (3)
O7—C22—C27—C26	175.3 (3)	C30—C29—O6—C23	−106.6 (3)
C25—C26—C27—C22	−1.2 (5)	C31—C29—O6—C23	133.3 (3)
C23—C24—C28—C29	9.1 (5)	C28—C29—O6—C23	12.4 (4)
C25—C24—C28—C29	−175.9 (4)	O8—C32—O7—C22	−13.8 (5)
C24—C28—C29—O6	−12.7 (5)	O9—C32—O7—C22	166.8 (2)
C24—C28—C29—C30	103.0 (4)	C23—C22—O7—C32	−63.1 (4)
C24—C28—C29—C31	−128.0 (4)	C27—C22—O7—C32	123.4 (3)
C38—C33—C34—O10	179.9 (3)	O8—C32—O9—C33	5.0 (5)
O9—C33—C34—O10	5.4 (4)	O7—C32—O9—C33	−175.6 (2)
C38—C33—C34—C35	0.4 (5)	C38—C33—O9—C32	57.3 (4)
O9—C33—C34—C35	−174.1 (3)	C34—C33—O9—C32	−128.3 (3)
O10—C34—C35—C36	178.9 (3)	C33—C34—O10—C40	168.4 (3)
C33—C34—C35—C36	−1.5 (5)	C35—C34—O10—C40	−12.0 (3)
O10—C34—C35—C39	−2.6 (4)	C41—C40—O10—C34	−97.2 (3)
C33—C34—C35—C39	177.1 (3)	C42—C40—O10—C34	142.3 (3)
C34—C35—C36—C37	1.1 (5)	C39—C40—O10—C34	20.9 (3)
C39—C35—C36—C37	−177.0 (4)	C43—C44—O11—C50	−170.8 (3)
C35—C36—C37—C38	0.5 (5)	C45—C44—O11—C50	9.4 (4)
C34—C33—C38—C37	1.2 (5)	C51—C50—O11—C44	104.7 (3)
O9—C33—C38—C37	175.4 (3)	C52—C50—O11—C44	−137.7 (3)
C36—C37—C38—C33	−1.6 (5)	C49—C50—O11—C44	−15.2 (3)
C36—C35—C39—C40	−166.5 (4)	O13—C53—O12—C43	3.5 (5)
C34—C35—C39—C40	15.2 (3)	O14—C53—O12—C43	−175.4 (3)
C35—C39—C40—O10	−21.6 (3)	C48—C43—O12—C53	−109.8 (4)
C35—C39—C40—C41	93.7 (3)	C44—C43—O12—C53	73.9 (4)
C35—C39—C40—C42	−136.8 (3)	O13—C53—O14—C54	1.4 (6)
C48—C43—C44—O11	−177.1 (3)	O12—C53—O14—C54	−179.6 (3)
O12—C43—C44—O11	−0.8 (5)	C55—C54—O14—C53	116.1 (4)
C48—C43—C44—C45	2.8 (5)	C59—C54—O14—C53	−70.7 (4)
O12—C43—C44—C45	179.0 (3)	C54—C55—O15—C61	−177.0 (3)
O11—C44—C45—C46	178.1 (3)	C56—C55—O15—C61	4.6 (4)
C43—C44—C45—C46	−1.7 (5)	C63B—C61—O15—C55	114.5 (7)
O11—C44—C45—C49	0.8 (4)	C62B—C61—O15—C55	−122.1 (6)
C43—C44—C45—C49	−179.0 (3)	C60—C61—O15—C55	−11.3 (4)
C44—C45—C46—C47	0.0 (5)	C63—C61—O15—C55	99.9 (7)
C49—C45—C46—C47	176.4 (4)	C62—C61—O15—C55	−152.9 (11)
